# Comprehensive Comparisons among Inotropic Agents on Mortality and Risk of Renal Dysfunction in Patients Who Underwent Cardiac Surgery: A Network Meta-Analysis of Randomized Controlled Trials

**DOI:** 10.3390/jcm10051032

**Published:** 2021-03-03

**Authors:** Wei-Cheng Chen, Meng-Hsuan Lin, Chieh-Lung Chen, Ying-Chieh Chen, Chih-Yu Chen, Yu-Chao Lin, Chin-Chuan Hung

**Affiliations:** 1 Graduate Institute of Biomedical Sciences, China Medical University, No. 91, Hsueh-Shih Road, Taichung 40402, Taiwan; pion501@gmail.com; 2 Division of Pulmonary and Critical Care Medicine, Department of Internal Medicine, China Medical University Hospital, No. 2, Yude Rd., North Dist., Taichung 404332, Taiwan; loyin1217@gmail.com (C.-L.C.); cychen0808@gmail.com (C.-Y.C.); 3 Department of Education, China Medical University Hospital, No. 2, Yude Rd., North Dist., Taichung 404332, Taiwan; 4 Department of Pharmacy, China Medical University, No. 100, Sec. 1, Jingmao Rd., Beitun Dist., Taichung 406040, Taiwan; becky19941114@gmail.com; 5 Department of Pharmacy, China Medical University Hsinchu Hospital, China Medical University, No. 199, Sec. 1, Xinglong Rd., Hsinchu County, Zhubei 30272, Taiwan; firenzejieh@gmail.com; 6 School of Medicine, China Medical University, No. 91, Hsueh-Shih Road, Taichung 40402, Taiwan; 7 Department of Pharmacy, China Medical University Hospital, No. 2 Yude Road, Taichung 40447, Taiwan; 8 Department of Healthcare Administration, Asia University, 500, Lioufeng Rd., Wufeng, Taichung 41354, Taiwan

**Keywords:** levosimendan, cardiac surgery, acute kidney injury, mortality, network meta-analysis

## Abstract

Several kinds of inotropes have been used in critically ill patients to improve hemodynamics and renal dysfunction after cardiac surgery; however, the treatment strategies for reducing mortality and increasing renal protection in patients who underwent cardiac surgery remain controversial. Therefore, we performed a comprehensive network meta-analysis to overcome the lack of head-to-head comparisons. A systematic database was searched up to 31 December 2020, for randomized controlled trials that compared different inotropes on mortality outcomes and renal protective effects after cardiac surgery. A total of 29 trials were included and a frequentist network meta-analysis was performed. Inconsistency analyses, publication bias, and subgroup analyses were also conducted. Compared with placebo, use of levosimendan significantly decreased the risks of mortality (odds ratio (OR): 0.74; 95% confidence interval (CI): 0.56–0.97) and risk of acute renal injury (OR: 0.61; 95% CI: 0.45–0.82), especially in low systolic function patients. Use of levosimendan also ranked the best treatment based on the P-score (90.1%), followed by placebo (64.5%), milrinone (49.6%), dopamine (49.5%), dobutamine (29.1%), and fenoldopam (17.0%). Taking all the available data into consideration, levosimendan was a safe renal-protective choice for the treatment of patients undergoing cardiac surgery, especially for those with low systolic function.

## 1. Introduction

Approximately 2 million cardiac surgeries are performed annually worldwide [1]. In 45% of patients undergoing cardiac surgery, renal failure, a major complication, occurs and is strongly associated with increased morbidity [2,3,4] and mortality [5,6,7,8]. Postoperative acute renal injury (AKI) is commonly due to inadequate tissue perfusion [9]. Besides low cardiac output and cardiogenic shock, post-cardiac surgery can precipitate renal failure, sepsis, vasoplegic shock, and atheroembolic etiologies of shock that can also cause AKI. However, treatments are different among low cardiac output syndrome (LCOS), sepsis, vasoplegic shock, and atheroembolic etiologies of shock-caused AKI. Inotropic agents are frequently administered in cardiac surgery patients to improve hemodynamic function to avoid hypotension and LCOS [10]. Therefore, the potentially renoprotective function of inotropes after cardiac surgery have been investigated [11,12].

Several types of inotropes are used clinically. Catecholamines remain the cornerstone of treatment for low cardiac output [11,12]. Through enhancement of the adrenergic pathway, they increase myocardial inotropy and chronotropy by binding to beta-1-adrenergic receptors, and they increase systemic vascular resistance by binding to alpha receptors. Unfortunately, a high degree of adrenergic stimulation can have pernicious effects during critical illness [13,14]. Phosphodiesterase 3 (PDE3) inhibitors, which are not altered by previous β-blockade, are indicated if catecholamines are ineffective. Because of their vasodilation effects, PDE3 inhibitors are less likely than dobutamine, a catecholamine, to increase heart rate and myocardial oxygen consumption [15]. Despite these advantages, myocardial ischemia and hypotensive episodes have been observed following their use [16]. Levosimendan, a calcium sensitizer and an ATP-sensitive potassium channel (KATP) opener, has properties of positive inotropy, vasodilation, and cardiac cytoprotection [17]. The cardioprotective effect of levosimendan and its active metabolite (OR1896) may be related to reductions in myocardial inflammation, remodeling, ischemia–reperfusion injury, and myocyte apoptosis [18,19,20,21]. Some meta-analyses, including some that have pooled the results of randomized controlled trials (RCTs), have concluded that levosimendan improves survival rate and exerts renoprotective effects in patients undergoing cardiac surgery [22,23,24,25,26,27,28,29,30]. By contrast, three large RCTs did not demonstrate these beneficial effects in patients with cardiovascular dysfunction [31,32,33]. The lack of a head-to-head comparison of inotropes may be a reason for the contradictory findings.

Network meta-analysis (NMA) is a robust statistical method that combines direct and indirect comparisons to solve the problem of missing data for head-to-head comparisons [34,35]. Accordingly, to evaluate the safety and efficacy of inotropes in patients undergoing cardiac surgery, we performed an NMA of RCTs to compare different pharmacological interventions in terms of mortality and renoprotective effects.

## 2. Materials and Methods

### 2.1. Search Strategy and Study Criteria

This protocol was registered and approved in the Prospective Register of Systematic Reviews, PROSPERO (CRD42020159411).

All relevant randomized controlled trials were searched from PubMed, EMBASE, Web of Science, and Cochrane Central Register of Controlled Trials (CENTRAL) up to December 2020, while some unpublished data were searched from ClinicalTrials.gov registers (clinicaltrials.gov, accessed on 31 December 2020). The keywords included the medical subject headings (MeSH) and text words for cardiac surgery and each treatment medication were as follows: cardiac surgery OR heart surgery AND levosimendan OR dopamine OR dobutamine OR milirinone AND kidney OR renal AND trial.

The study abstracts were used to filter the results obtained from e-databases; relevant studies were then collected as full-text articles. Eligible studies meeting the following criteria were included: (1) randomized controlled trials reporting on mortality or renal-related endpoints; (2) enrolled patients aged 18 years or older with acute preoperative circulatory insufficiency requiring treatment with a positive inotrope or by mechanical means (IABP or ECLS); (3) intervention group received any inotropic agents, such as catecholamines, phosphodiesterase-3 inhibitors, or other calcium sensitizer drug, such as levosimendan; (4) comparators were placebo or any inotropic agents other than the agent used in the intervention group; (5) study reports were not limited by language; and (6) the cardiac surgical procedure could be coronary artery bypass grafting (CABG), CABG plus aortic valve surgery, isolated mitral valve surgery, or any combination of these procedures. However, the exclusion criteria were as following: (1) patients with cardiogenic shock, sepsis, vasoplegic shock, or atheroembolic etiologies of shock; (2) patients with severe preoperative renal insufficiency (Scr > 1.5 mg/dL); and (3) studies that included cardiotonic agents that were used less frequently, i.e., dopexamine, enoximone and amrinone.

### 2.2. Data Extraction and Risk of Bias Assessment

The primary outcome was the longest following-up mortality after cardiac surgery with the treatment of inotropes. The secondary outcomes were established as renal-related problems, such as the incidence of acute kidney injury (AKI), renal replacement therapy (RRT)—including hemodialysis, peritoneal dialysis, or continuous venovenous hemodialysis—and time in an intensive care unit (ICU) during the use of an inotropic agent after cardiac surgery.

The information collected from each article included: primary outcome and secondary outcomes, cardiac surgery setting details, sample size, the inotropic agent’s dosing regimen, patients’ baseline heart and renal function, and acute kidney injury definition if data were available. In addition, a Cochrane risk-of-bias tool (version 5.1.0, Cochrane, London, UK) was applied to evaluate the risk of bias of included trials.

All of the above were performed by two independent investigators. Furthermore, if any disagreement between them occurred, a consensus was reached with a third reviewer.

### 2.3. Statistical Analysis

Network meta-analysis, which is a multiple treatment analysis method that combines direct and indirect evidence, makes it feasible to contrast treatments that would be unable to be compared by a pairwise approach. In addition, our network meta-analysis used a frequentist random effects model and reported results on the basis of the Preferred Reporting Items for Systematic Reviews and Meta-Analyses (PRISMA) guidelines.

For discrete data such as mortality, AKI, and RRT, we used an odds ratio (OR) with 95% confidence intervals (CIs) calculated by the Mantel–Haenszel method to measure the endpoints of study. Mean difference (MD) with 95% CI was used with outcomes involving continuous data, such as ICU length of stay. If the mean and standard deviation (SD) were not given, other values such as the median and interquartile range (IQR) were converted to means and SDs to obtain a mean difference [36].

Speculating on the heterogeneity among the intervention effects, we used a random effects model on the inverse-variance weights according to the DerSimonian–Laird method to achieve more unbiased statistics. Local inconsistency analyses were demonstrated by a loop-specific approach that evaluated incoherence separately in each closed loop between direct and indirect arms (*p*-values < 0.05 mean inconsistencies) to assure that there were no discrepancies and to appraise the robustness of the network meta-analysis. A funnel plot was used to assess the publication bias.

Rank probability, the probability that an intervention was at a specific rank when compared with the other interventions, was applied in network as P-score. Between multi-arm tests, the P-score enhanced the precise effect of comparisons and consideration of the correlation. This rating estimated from highest to lowest the probability of each outcome for each drug contrasted to placebo. A P-score can range from 0% (i.e., this treatment was the lowest rank) to 100% (i.e., this treatment was the highest rank).

Pooled data using network meta-analysis were analyzed by R version 3.5.1 with the netmeta package (https://CRAN.R-project.org/package=netmeta, accessed on 12 January 2021). In addition, if a network indicated that a specific drug had both safety and renal-related efficacy, we performed a subgroup analysis (a *p*-value of the subgroup difference less than 0.1 indicated a statistically significant subgroup effect) to elucidate the exact clinical settings by Revman 5.3. (Cochrane collaboration, Copenhagen: The Nordic Cochrane Centre, The Cochrane Collaboration, 2014, London, UK).

## 3. Results

### 3.1. Study Enrollment

**Figure 1:**
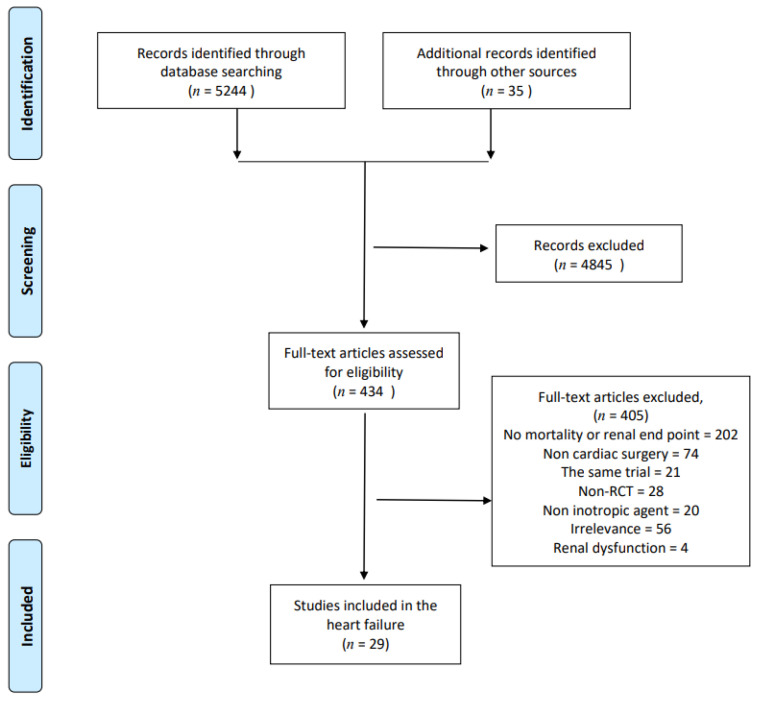
Preferred Reporting Items for Systematic Reviews and Meta-Analyses (PRISMA) flow diagram of randomized controlled trials included and excluded. Non-RCT: none randomized controlled trials.

Finally, a total of 434 articles which were screened from 5279 references were evaluated by two independent researchers. Papers were excluded for the following reasons: no outcome data including mortality or renal end point (202 studies), non-cardiac surgery (74 studies), multiple reports of the same trial (21 studies), non-randomized controlled trials (28 studies), no use of an inotropic agent (20 studies), irrelevant papers (56 studies), and renal dysfunction studies (4 studies). Therefore, 29 RCTs were included, with which we conducted the network meta-analysis (Figure 1).

### 3.2. Characteristics of Included Trials

Characteristics of these trials are listed in Supplementary Table S1. This study analyzed a total of five inotropic agents: levosimendan, milrinone, dobutamine, dopamine, and fenoldopam.

In addition, 28 trials reported outcomes on mortality [31,32,33,37,38,39,40,41,42,43,44,45,46,47,48,49,50,51,52,53,54,55,56,57,58,59,60,61] (3641 patients), 16 trials reported the incidence of acute kidney injury [32,33,37,39,40,44,47,49,51,52,54,55,56,58,61,62] (2678 patients), 14 trials reported on the need of renal replacement therapy [31,32,33,40,41,44,46,47,49,50,52,53,54,55] (2923 patients), and 15 trials reported on the length of ICU stay [31,32,33,37,40,42,44,52,53,55,56,58,60,61,62] (2504 patients). Furthermore, we established four networks, one for each endpoint under analysis (Supplementary Materials Figure S1).

### 3.3. Risk of Bias

Most of the studies were of moderate to high quality. A total of 23 (61%) studies were judged to carry a low risk of bias [31,32,33,37,39,47,50,51,52,53,56,58,59,60,61,62], 10 (26%) a moderate risk of bias [38,40,42,43,45,48,49,55], and 5 (13%) a high risk of bias [41,44,46,54,57]. The most common bias was non-blinded study bias in about 32% of included studies with an open-label design. Detail on the quality assessment of each study is depicted in Supplementary Figure S2.

### 3.4. Mortality

The network analysis showed that use of levosimendan significantly reduced mortality compared to placebo (OR: 0.74; 95% CI: 0.56–0.97) (Table 1). On the other hand, use of dobutamine significantly increased mortality compared to placebo (OR: 1.98; 95% CI: 1.01–3.91) and levosimendan (OR: 2.69; 95% CI: 1.44–5.01).

### 3.5. Acute Kidney Injury (AKI)

Pooled analysis of all studies found that use of levosimendan (OR: 0.61; 95% CI: 0.45–0.82) was associated with significantly lower AKI incidence compared to placebo (Table 1). In addition, use of dobutamine resulted in significantly higher AKI incidence compared to fenoldopam (OR: 34.81; 95% CI: 1.49–811.85), levosimendan (OR: 5.72; 95% CI: 2.01–16.27), and placebo (OR: 3.49; 95% CI: 1.17–10.34).

### 3.6. Renal Replacement Therapy (RRT)

The network analysis showed that use of dobutamine (OR: 3.65; 95% CI: 1.41–9.46) was associated with significantly higher RRT risk compared to levosimendan (Table 2).

### 3.7. ICU Stay

The network analysis showed that use of levosimendan significantly reduced ICU stay time compared to placebo (MD: −0.55; 95% CI: −1.00 to −0.09) (Table 2), while use of dobutamine significantly increased ICU length of stay compared to dopamine (MD: −3.48; 95% CI: 0.61–6.63), levosimendan (MD: −3.83; 95% CI: 2.72–4.94), milrinone (MD:−3.37; 95% CI: 2.04–4.70), and placebo (MD: −3.28; 95% CI: 2.09–4.48).

### 3.8. Finding of Ranking

**Figure 2:**
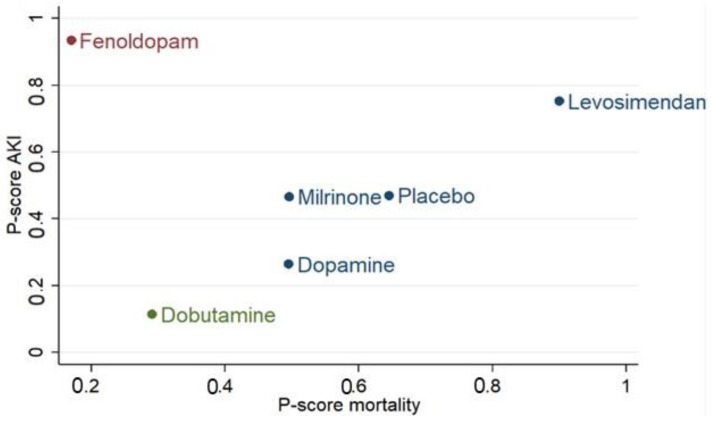
P-scores ranking plot. P-scores ranking plot represents the mortality and the acute kidney injury (AKI) of interventions in a cardiac surgery population. Treatment with better efficacy should be in the upper-right corner of the graph.

In a ranking analysis performed with P-scores, the use of levosimendan ranked the highest in mortality (P-score: 0.90), RRT (P-score: 0.90), and ICU length of stay (P-score: 0.87) among the included inotropic agents (Supplementary Table S2). Though use of fenoldopam exhibited a superior P-score in AKI, it seemed to be associated with an increase in death. The sum of ranking finding for safety (P-score mortality) and renal protective efficacy (P-score AKI) is presented together in a bivariate ranking plot (Figure 2). To sum up, the results demonstrate that levosimendan had a better effect than others.

### 3.9. Inconsistency Analyses

According to the direct and indirect estimations, no further differences were found (Supplementary Table S3).

### 3.10. Publication Bias

No significant publication bias (Egger’s test, *p* > 0.05) of any relevant outcomes were identified (Supplementary Figure S3).

### 3.11. Subgroup Analyses

In addition to the principal network meta-analyses, we also performed subgroup analyses to investigate specific clinical settings of levosimendan that had a significant effect on reducing mortality and AKI. The following were the predefined subgroup analyses for mortality and AKI: (1) Systolic function: Trials that administered levosimendan to patients with preoperative systolic function (low left ventricular ejection fraction (LVEF), cardiac index (CI), and low cardiac output syndrome (LCOS), or preserved LVEF and CI); (2) Administration timing: Trials that administered levosimendan preoperatively versus intraoperatively versus postoperatively; and (3) Operation method: Trials that administered levosimendan under different operation method, CABG versus valve versus all kinds of cardiac surgery.

**Figure 3:**
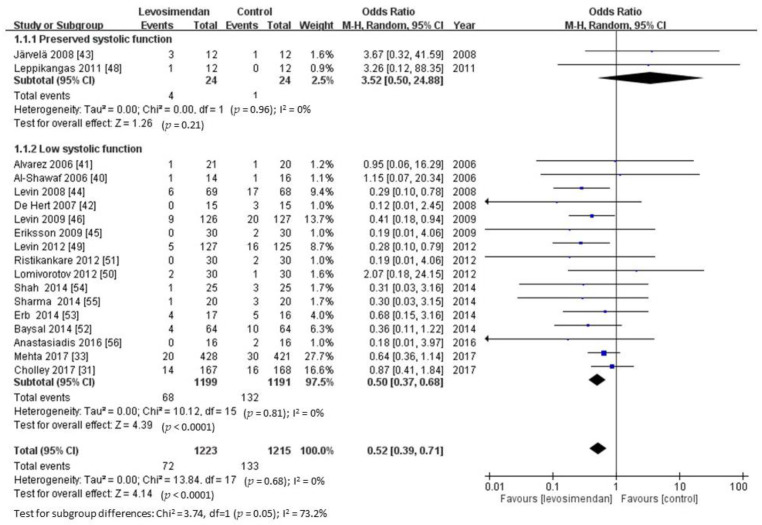
Subgroup analyses for mortality by systolic function. Data presented as odd ratio (OR) with 95% confidence interval (CI). *p*-value of subgroup difference <0.1 means a significant subgroup effect.

Only on mortality outcome did use of levosimendan show a significant subgroup effect (*p*-value of subgroup difference <0.1), meaning that systolic function significantly modified the effect of levosimendan in comparison to control (Figure 3). Levosimendan was significantly favored over control for low systolic function, while control was favored over levosimendan for high systolic function; therefore, the subgroup effect was qualitative.

Even though other subgroup analyses showed non-overlap of confidence intervals (ostensibly indicating a statistically significant difference), only specific tests of subgroup difference showed subgroup effects. Consequently, neither mortality nor AKI had a subgroup effect for administration timing or operation method (Supplementary Figures S4–S8).

## 4. Discussion

The present NMA comprehensively compared the mortality and renoprotective effects of all inotropes used in cardiac surgery. Levosimendan significantly reduced mortality, length of ICU stay, and AKI risk. Although fenoldopam exhibited a superior P-score in AKI, it also seemed to be associated with increased mortality.

Most meta-analyses have reported an integrated useful effect of levosimendan on survival rate [22,23,24,25,26,27,28] and renal protection compared with other treatment regimens in patients undergoing cardiac surgery [29,30], but three recent large multicenter RCTs (LICORN [31], CHEETAH [32], and LEVO-CTS [33]) found neutral or inconclusive results. This discrepancy between the results of the three large RCTs and previous small trials may be due to the following reasons [63]: (1) The patients in the three RCTs received a relatively low dose of levosimendan without bolus, which may have prevented potent vasodilatation and consequent hypotension but also caused loss of noticeable hemodynamic effects; (2) In the LEVO-CTS [33] and LICORN [31] trials, levosimendan was started very shortly before surgery, which may have limited the preconditioning effect; and (3) In the CHEETAH [32] trial, pretreatment with high doses of beta-mimetic drugs may have reduced the inotropic effect of levosimendan [64]. Although small-study effects are a known problem in meta-analysis, small studies can also show larger treatment effects [65] because they have more controlled situations than a multicenter trial does. In other words, our study evaluated every trial equally regardless of its size [66].

In the present NMA, levosimendan use did not result in a reduction rate of RRT in patients undergoing cardiac surgery, which was consistent with the three large RCTs. Although the use of RRT constituted relatively clear criteria, this outcome occurred in only 1–2% of patients, indicating that the choice of end point remained a critical challenge. However, our data also indicated a benefit of levosimendan in terms of mortality and AKI. Preclinical experiments have demonstrated the renoprotective effect of levosimendan [67,68,69]. Levosimendan may improve renal filtration by selective afferent arteriolar vasodilation [70]. Furthermore, to optimize the safety and efficacy of levosimendan, the specific patient groups that may benefit the most from its use must be determined. As suggested in our study and in previous meta-analyses [26,33], patients with low baseline systolic function exhibited a significant reduction in mortality rate compared with controls. Moreover, in contrast to the on–off action of other inotropes, the prolonged effect of levosimendan, which lasts for several days [42,71], might be a major reason for its use.

Our finding that dobutamine use is associated with poorer outcomes is consistent with previous research. An observational study reported that dobutamine administration was associated with major cardiac morbidity [72]. Postoperative inotrope use, including epinephrine, milrinone, and dobutamine, is independently associated with increased in-hospital mortality and renal dysfunction [73]. However, despite the potential harmful effects of catecholamines, inotropes are routinely prescribed to patients with low cardiac output syndrome after cardiac surgery, according to current practice guidelines [74].

Our study has several strengths. First, extensive literature searches were conducted and related RCTs were included. Second, we included only RCTs in the NMA; thus, our results are of reasonable quality. Furthermore, comprehensive indirect comparisons were performed among different inotropes on subgroup sets of clinical and renal physiologic outcomes. Nevertheless, our study also has several limitations. First, to address the heterogeneity of inclusion criteria, most included studies had patients with normal preoperative renal function. Therefore, our results may not be generalizable to patients with preexisting renal dysfunction. However, pooling data with similar but not identical renal outcome criteria might have introduced bias, potentially influencing our results. Second, the covariate distribution was uneven in subgroup analyses. Because of inadequate number of studies, subgroup analyses might result in confounding. Specifically, the selection of study characteristics for subgroup analysis was challenging because of limited definitions in each study. To maintain the original design of each study, we used inclusion criteria instead of the average systolic function at baseline. Our data revealed a significant subgroup difference only in systolic function on mortality outcome. However, the result should be interpreted cautiously because a much smaller number of trials and participants contributed data to the low systolic function subgroup than to the high systolic function subgroup. Consequently, the limited number of articles in the subgroup analysis may have caused broad eligibility criteria and imbalanced distribution of effect modifiers; further trials for precise estimation are required. Third, many included studies had a moderate to high risk of bias, the most common being lack of blinding. However, the influence of blinding varies depending on the type of outcome. For instance, in our NMA, overestimation was unlikely when the outcome was objectively measured (e.g., death). Some earlier studies might have been judged as having unclear bias due to limited information. However, to evaluate the holistic effects of inotropic agents, we did not rule out these early studies. Fourth, we investigated a mixed population of patients who were undergoing different cardiac surgical operations, including a few patients without cardiopulmonary bypass. Therefore, we performed subgroup analysis to deal with this limitation. The results demonstrate that there was no subgroup difference and suggest an equal benefit with levosimendan treatment in different operation methods (Supplementary Figures S4–S7).

## 5. Conclusions

In summary, taking all the available network meta-analysis data into consideration, levosimendan may be a safe renal-protective inotropic agent for the treatment of patients undergoing cardiac surgery. Further in-depth evaluation of the use of levosimendan requires additional trials in well-defined patient populations, and its study design should minimize the impact of patient management.

## Figures and Tables

**Table 1:** Results from the multiple-treatment comparison analyses for mortality and acute kidney injury (AKI).

AKI OR (95% CI) ^#^					
Dobutamine	1.19 (0.04–35.96)	34.81 (1.49–811.85) *	5.72 (2.01–16.27) *	3.2 (0.63–16.33)	3.49 (1.17–10.34) *
1.36 (0.13–14.05)	Dopamine	29.24 (0.37–2329.85)	4.8 (0.19–123.11)	2.69 (0.08–85.69)	2.93 (0.12–73.99)
0.46 (0.04–4.74)	0.34 (0.01–7.97)	Fenoldopam	0.16 (0.01–3.21)	0.09 (0–2.27)	0.1 (0.01–1.92)
2.69 (1.44–5.01) *	1.98 (0.21–18.83)	5.88 (0.62–55.97)	Levosimendan	0.56 (0.16–1.95)	0.61 (0.45–0.82) *
1.45 (0.39–5.32)	1.06 (0.09–13.05)	3.16 (0.26–38.78)	0.54 (0.17–1.69)	Milrinone	1.09 (0.31–3.8)
1.98 (1.01–3.91) *	1.46 (0.16–13.66)	4.33 (0.46–40.61)	0.74 (0.56–0.97) *	1.37 (0.44–4.25)	Placebo
Mortality OR (95% CI) ^#^					

Comparisons between treatments are read from left to right, and the estimate with 95% confidence interval for a given comparison is read at the intersection of two treatments. ^#^ Numeric values of this outcome are presented as an odds ratio (OR) with 95% confidence interval (CI). An OR smaller than 1 favors the column-defined treatment, while an OR higher than 1 favors the row-defined treatment. * Denotes *p*-value < 0.05.

**Table 2:** Results from the multiple-treatment comparison analyses for renal replacement therapy (RRT) and intensive care unit (ICU).

RRT OR (95% CI) ^#^				
Dobutamine	−	3.65 (1.41–9.46) *	1.30 (0.04–39.54)	2.63 (0.96–7.21)
3.48 (0.61–6.36) *	Dopamine	−	−	−
3.83 (2.72–4.94) *	0.35 (−2.31–3.00)	Levosimendan	0.36 (0.01–9.47)	0.72 (0.52–1.00)
3.37 (2.04–4.70) *	−0.12 (−2.79–2.56)	−0.46 (−1.20–0.27)	Milrinone	2.03 (0.08–54.70)
3.28 (2.09–4.48) *	−0.20 (−2.81–2.41)	−0.55 (−1.00−0.09) *	−0.08 (−0.66–0.50)	Placebo
ICU MD (95% CI) ^+^				

Comparisons between treatments are read from left to right, and the estimate with 95% confidence interval for a given comparison is read at the intersection of two treatments. ^#^ Numeric values of this outcome are presented as an odds ratio (OR) with 95% confidence interval (CI). An OR smaller than 1 favors the column-defined treatment, while an OR higher than 1 favors row-defined treatment. ^+^ Values of this outcome are presented as mean difference (MD) with 95% confidence interval (CI). An MD smaller than 0 favors the column-defined treatment, while an MD higher than 0 favors the row-defined treatment. * Denotes *p*-value < 0.05.

## Data Availability

The datasets used and/or analyzed during the current study are available from the corresponding author on reasonable request.

## Supplementary Materials

The following are available online at https://www.mdpi.com/2077-0383/10/5/1032/s1. Table S1: Detailed characteristics of each included study, Table S2: P-score analyses for the evaluated outcomes, Table S3: Consistency analyses of each outcome by node-split model, Figure S1: Network plot for included therapies, Figure S2: Risk of bias, Figure S3: Funnel plot for each outcome, Figure S4: Subgroup analyses for mortality by administration timing, Figure S5: Subgroup analyses for mortality by operation method, Figure S6: Subgroup analyses for AKI by systolic function, Figure S7: Subgroup analyses for AKI by administration timing, Figure S8: Subgroup analyses for AKI by operation method.

## References

[1] Parikh C.R., Coca S.G., Thiessen-Philbrook H., Shlipak M.G., Koyner J.L., Wang Z., Edelstein C.L., Devarajan P., Patel U.D., Zappitelli M. (2011). Postoperative biomarkers predict acute kidney injury and poor outcomes after adult cardiac surgery. J. Am. Soc. Nephrol. JASN.

[2] Ryckwaert F., Boccara G., Frappier J.M., Colson P.H. (2002). Incidence, risk factors, and prognosis of a moderate increase in plasma creatinine early after cardiac surgery. Crit. Care Med..

[3] Stafford-Smith M., Podgoreanu M., Swaminathan M., Phillips-Bute B., Mathew J.P., Hauser E.H., Winn M.P., Milano C., Nielsen D.M., Smith M. (2005). Association of genetic polymorphisms with risk of renal injury after coronary bypass graft surgery. Am. J. Kidney Dis. Off. J. Natl. Kidney Found..

[4] Crawford T.C., Magruder J.T., Grimm J.C., Suarez-Pierre A., Sciortino C.M., Mandal K., Zehr K.J., Conte J.V., Higgins R.S., Cameron D.E. (2017). Complications after Cardiac Operations: All Are Not Created Equal. Ann. Thorac. Surg..

[5] Brown J.R., Cochran R.P., Dacey L.J., Ross C.S., Kunzelman K.S., Dunton R.F., Braxton J.H., Charlesworth D.C., Clough R.A., Helm R.E. (2006). Perioperative increases in serum creatinine are predictive of increased 90-day mortality after coronary artery bypass graft surgery. Circulation.

[6] Koyner J.L., Bennett M.R., Worcester E.M., Ma Q., Raman J., Jeevanandam V., Kasza K.E., O’Connor M.F., Konczal D.J., Trevino S. (2008). Urinary cystatin C as an early biomarker of acute kidney injury following adult cardiothoracic surgery. Kidney Int..

[7] Hobson C.E., Yavas S., Segal M.S., Schold J.D., Tribble C.G., Layon A.J., Bihorac A. (2009). Acute kidney injury is associated with increased long-term mortality after cardiothoracic surgery. Circulation.

[8] Zimmerman R.F., Ezeanuna P.U., Kane J.C., Cleland C.D., Kempananjappa T.J., Lucas F.L., Kramer R.S. (2011). Ischemic preconditioning at a remote site prevents acute kidney injury in patients following cardiac surgery. Kidney Int..

[9] Thakar C.V. (2013). Perioperative acute kidney injury. Adv. Chronic Kidney Dis..

[10] Engelman D.T., Ben Ali W., Williams J.B., Perrault L.P., Reddy V.S., Arora R.C., Roselli E.E., Khoynezhad A., Gerdisch M., Levy J.H. (2019). Guidelines for Perioperative Care in Cardiac Surgery: Enhanced Recovery After Surgery Society Recommendations. JAMA Surg..

[11] Lomivorotov V.V., Efremov S.M., Kirov M.Y., Fominskiy E.V., Karaskov A.M. (2017). Low-Cardiac-Output Syndrome After Cardiac Surgery. J. Cardiothorac. Vasc. Anesth..

[12] Gillies M., Bellomo R., Doolan L., Buxton B. (2005). Bench-to-bedside review: Inotropic drug therapy after adult cardiac surgery—A systematic literature review. Crit. Care.

[13] Dünser M.W., Hasibeder W.R. (2009). Sympathetic overstimulation during critical illness: Adverse effects of adrenergic stress. J. Intensive Care Med..

[14] Schmittinger C.A., Torgersen C., Luckner G., Schröder D.C., Lorenz I., Dünser M.W. (2012). Adverse cardiac events during catecholamine vasopressor therapy: A prospective observational study. Intensive Care Med..

[15] Grose R., Strain J., Greenberg M., LeJemtel T.H. (1986). Systemic and coronary effects of intravenous milrinone and dobutamine in congestive heart failure. J. Am. Coll. Cardiol..

[16] Chong L.Y.Z., Satya K., Kim B., Berkowitz R. (2018). Milrinone Dosing and a Culture of Caution in Clinical Practice. Cardiol. Rev..

[17] Papp Z., Édes I., Fruhwald S., De Hert S.G., Salmenperä M., Leppikangas H., Mebazaa A., Landoni G., Grossini E., Caimmi P. (2012). Levosimendan: Molecular mechanisms and clinical implications: Consensus of experts on the mechanisms of action of levosimendan. Int. J. Cardiol..

[18] Maytin M., Colucci W.S. (2005). Cardioprotection: A new paradigm in the management of acute heart failure syndromes. Am. J. Cardiol..

[19] Louhelainen M., Vahtola E., Kaheinen P., Leskinen H., Merasto S., Kytö V., Finckenberg P., Colucci W.S., Levijoki J., Pollesello P. (2007). Effects of levosimendan on cardiac remodeling and cardiomyocyte apoptosis in hypertensive Dahl/Rapp rats. Br. J. Pharm..

[20] Grossini E., Caimmi P.P., Platini F., Molinari C., Uberti F., Cattaneo M., Valente G., Mary D.A., Vacca G., Tessitore L. (2010). Modulation of programmed forms of cell death by intracoronary levosimendan during regional myocardial ischemia in anesthetized pigs. Cardiovasc. Drugs Ther..

[21] Caimmi P.P., Molinari C., Uberti F., Micalizzi E., Valente G., Mary D.A., Vacca G., Grossini E. (2011). Intracoronary levosimendan prevents myocardial ischemic damages and activates survival signaling through ATP-sensitive potassium channel and nitric oxide. Eur. J. Cardio-Thorac. Surg. Off. J. Eur. Assoc. Cardio-Thorac. Surg..

[22] Belletti A., Castro M.L., Silvetti S., Greco T., Biondi-Zoccai G., Pasin L., Zangrillo A., Landoni G. (2015). The Effect of inotropes and vasopressors on mortality: A meta-analysis of randomized clinical trials. Br. J. Anaesth..

[23] Thackray S., Easthaugh J., Freemantle N., Cleland J.G. (2002). The effectiveness and relative effectiveness of intravenous inotropic drugs acting through the adrenergic pathway in patients with heart failure-a meta-regression analysis. Eur. J. Heart Fail..

[24] Landoni G., Mizzi A., Biondi-Zoccai G., Bruno G., Bignami E., Corno L., Zambon M., Gerli C., Zangrillo A. (2010). Reducing mortality in cardiac surgery with levosimendan: A meta-analysis of randomized controlled trials. J. Cardiothorac. Vasc. Anesth..

[25] Landoni G., Biondi-Zoccai G., Greco M., Greco T., Bignami E., Morelli A., Guarracino F., Zangrillo A. (2012). Effects of levosimendan on mortality and hospitalization. A meta-analysis of randomized controlled studies. Crit. Care Med..

[26] Harrison R.W., Hasselblad V., Mehta R.H., Levin R., Harrington R.A., Alexander J.H. (2013). Effect of levosimendan on survival and adverse events after cardiac surgery: A meta-analysis. J. Cardiothorac. Vasc. Anesth..

[27] Greco T., Calabrò M.G., Covello R.D., Greco M., Pasin L., Morelli A., Landoni G., Zangrillo A. (2015). A Bayesian network meta-analysis on the effect of inodilatory agents on mortality. Br. J. Anaesth..

[28] Lee C.T., Lin Y.C., Yeh Y.C., Chen T.L., Chen C.Y. (2017). Effects of levosimendan for perioperative cardiovascular dysfunction in patients receiving cardiac surgery: A meta-analysis with trial sequential analysis. Intensive Care Med..

[29] Bove T., Matteazzi A., Belletti A., Paternoster G., Saleh O., Taddeo D., Dossi R., Greco T., Bradic N., Husedzinovic I. (2015). Beneficial impact of levosimendan in critically ill patients with or at risk for acute renal failure: A meta-analysis of randomized clinical trials. Heart Lung Vessel..

[30] Zhou C., Gong J., Chen D., Wang W., Liu M., Liu B. (2016). Levosimendan for Prevention of Acute Kidney Injury After Cardiac Surgery: A Meta-analysis of Randomized Controlled Trials. Am. J. Kidney Dis. Off. J. Natl. Kidney Found..

[31] Cholley B., Caruba T., Grosjean S., Amour J., Ouattara A., Villacorta J., Miguet B., Guinet P., Levy F., Squara P. (2017). Effect of Levosimendan on Low Cardiac Output Syndrome in Patients with Low Ejection Fraction Undergoing Coronary Artery Bypass Grafting With Cardiopulmonary Bypass: The LICORN Randomized Clinical Trial. JAMA.

[32] Landoni G., Lomivorotov V.V., Alvaro G., Lobreglio R., Pisano A., Guarracino F., Calabro M.G., Grigoryev E.V., Likhvantsev V.V., Salgado-Filho M.F. (2017). Levosimendan for Hemodynamic Support after Cardiac Surgery. N. Engl. J. Med..

[33] Mehta R.H., Leimberger J.D., van Diepen S., Meza J., Wang A., Jankowich R., Harrison R.W., Hay D., Fremes S., Duncan A. (2017). Levosimendan in Patients with Left Ventricular Dysfunction Undergoing Cardiac Surgery. N. Engl. J. Med..

[34] Salanti G. (2012). Indirect and mixed-treatment comparison, network, or multiple-treatments meta-analysis: Many names, many benefits, many concerns for the next generation evidence synthesis tool. Res. Synth. Methods.

[35] Tonin F.S., Rotta I., Mendes A.M., Pontarolo R. (2017). Network meta-analysis: A technique to gather evidence from direct and indirect comparisons. Pharm. Pract..

[36] Wan X., Wang W., Liu J., Tong T. (2014). Estimating the sample mean and standard deviation from the sample size, median, range and/or interquartile range. BMC Med. Res. Methodol..

[37] Lassnigg A., Donner E., Grubhofer G., Presterl E., Druml W., Hiesmayr M. (2000). Lack of renoprotective effects of dopamine and furosemide during cardiac surgery. J. Am. Soc. Nephrol. JASN.

[38] Woo E.B., Tang A.T., el-Gamel A., Keevil B., Greenhalgh D., Patrick M., Jones M.T., Hooper T.L. (2002). Dopamine therapy for patients at risk of renal dysfunction following cardiac surgery: Science or fiction?. Eur. J. Cardio-Thorac. Surg. Off. J. Eur. Assoc. Cardio-Thorac. Surg..

[39] Ranucci M., De Benedetti D., Bianchini C., Castelvecchio S., Ballotta A., Frigiola A., Menicanti L. (2010). Effects of fenoldopam infusion in complex cardiac surgical operations: A prospective, randomized, double-blind, placebo-controlled study. Minerva Anestesiol..

[40] Al-Shawaf E., Ayed A., Vislocky I., Radomir B., Dehrab N., Tarazi R. (2006). Levosimendan or milrinone in the type 2 diabetic patient with low ejection fraction undergoing elective coronary artery surgery. J. Cardiothorac. Vasc. Anesth..

[41] Álvarez J., Bouzada M., Fernández Á.L., Caruezo V., Taboada M., Rodríguez J., Ginesta V., Rubio J., García-Bengoechea J.B., González-Juanatey J.R. (2006). Comparación de los efectos hemodinámicos del levosimendán con la dobutamina en pacientes con bajo gasto después de cirugía cardiaca. Rev. Española Cardiol..

[42] De Hert S.G., Lorsomradee S., Cromheecke S., Van der Linden P.J. (2007). The effects of levosimendan in cardiac surgery patients with poor left ventricular function. Anesth. Analg..

[43] Jarvela K., Maaranen P., Sisto T., Ruokonen E. (2008). Levosimendan in aortic valve surgery: Cardiac performance and recovery. J. Cardiothorac. Vasc. Anesth..

[44] Levin R.L., Degrange M.A., Porcile R., Salvagio F., Blanco N., Botbol A.L., Tanus E., del Mazo C.D. (2008). The calcium sensitizer levosimendan gives superior results to dobutamine in postoperative low cardiac output syndrome. Rev. Esp. Cardiol..

[45] Eriksson H.I., Jalonen J.R., Heikkinen L.O., Kivikko M., Laine M., Leino K.A., Kuitunen A.H., Kuttila K.T., Perakyla T.K., Sarapohja T. (2009). Levosimendan facilitates weaning from cardiopulmonary bypass in patients undergoing coronary artery bypass grafting with impaired left ventricular function. Ann. Thorac. Surg..

[46] Levin R., Rafael Porcile R., Salvagio F. (2009). Levosimendan reduces mortality in postoperative low cardiac output syndrome after coronary surgery. Circulation.

[47] Lahtinen P., Pitkanen O., Polonen P., Turpeinen A., Kiviniemi V., Uusaro A. (2011). Levosimendan reduces heart failure after cardiac surgery: A prospective, randomized, placebo-controlled trial. Crit. Care Med..

[48] Leppikangas H., Jarvela K., Sisto T., Maaranen P., Virtanen M., Lehto P., Karlsson S., Koobi T., Lindgren L. (2011). Preoperative levosimendan infusion in combined aortic valve and coronary bypass surgery. Br. J. Anaesth..

[49] Levin R., Degrange M., Del Mazo C., Tanus E., Porcile R. (2012). Preoperative levosimendan decreases mortality and the development of low cardiac output in high-risk patients with severe left ventricular dysfunction undergoing coronary artery bypass grafting with cardiopulmonary bypass. Exp. Clin. Cardiol..

[50] Lomivorotov V.V., Boboshko V.A., Efremov S.M., Kornilov I.A., Chernyavskiy A.M., Lomivorotov V.N., Knazkova L.G., Karaskov A.M. (2012). Levosimendan versus an intra-aortic balloon pump in high-risk cardiac patients. J. Cardiothorac. Vasc. Anesth..

[51] Ristikankare A., Poyhia R., Eriksson H., Valtonen M., Leino K., Salmenpera M. (2012). Effects of levosimendan on renal function in patients undergoing coronary artery surgery. J. Cardiothorac. Vasc. Anesth..

[52] Baysal A., Yanartas M., Dogukan M., Gundogus N., Kocak T., Koksal C. (2014). Levosimendan improves renal outcome in cardiac surgery: A randomized trial. J. Cardiothorac. Vasc. Anesth..

[53] Erb J., Beutlhauser T., Feldheiser A., Schuster B., Treskatsch S., Grubitzsch H., Spies C. (2014). Influence of levosimendan on organ dysfunction in patients with severely reduced left ventricular function undergoing cardiac surgery. J. Int. Med. Res..

[54] Shah B., Sharma P., Brahmbhatt A., Shah R., Rathod B., Shastri N., Patel J., Malhotra A. (2014). Study of levosimendan during off-pump coronary artery bypass grafting in patients with LV dysfunction: A double-blind randomized study. Indian J. Pharm..

[55] Sharma P., Malhotra A., Gandhi S., Garg P., Bishnoi A., Gandhi H. (2014). Preoperative levosimendan in ischemic mitral valve repair. Asian Cardiovasc Thorac Ann..

[56] Anastasiadis K., Antonitsis P., Vranis K., Kleontas A., Asteriou C., Grosomanidis V., Tossios P., Argiriadou H. (2016). Effectiveness of prophylactic levosimendan in patients with impaired left ventricular function undergoing coronary artery bypass grafting: A randomized pilot study. Interact. Cardiovasc Thorac. Surg..

[57] Shi Y., Denault A.Y., Couture P., Butnaru A., Carrier M., Tardif J.C. (2006). Biventricular diastolic filling patterns after coronary artery bypass graft surgery. J. Thorac. Cardiovasc. Surg..

[58] Couture P., Denault A.Y., Pellerin M., Tardif J.C. (2007). Milrinone enhances systolic, but not diastolic function during coronary artery bypass grafting surgery. Can. J. Anaesth. J. Can. D’Anesthesie.

[59] Arbeus M., Axelsson B., Friberg O., Magnuson A., Bodin L., Hultman J. (2009). Milrinone increases flow in coronary artery bypass grafts after cardiopulmonary bypass: A prospective, randomized, double-blind, placebo-controlled study. J. Cardiothorac. Vasc. Anesth..

[60] Jebeli M., Ghazinoor M., Mandegar M.H., Rasouli M.R., Eghtesadi-Araghi P., Goodarzynejad H., Mohammadzadeh R., Darehzereshki A., Dianat S. (2010). Effect of milrinone on short-term outcome of patients with myocardial dysfunction undergoing coronary artery bypass graft: A randomized controlled trial. Cardiol. J..

[61] Hadadzadeh M., Hosseini S.H., Mostafavi Pour Manshadi S.M., Naderi N., Emami Meybodi M. (2013). Effect of milrinone on short term outcome of patients with myocardial dysfunction undergoing off-pump coronary artery bypass graft: A randomized clinical trial. Acta Med. Iran..

[62] Tritapepe L., De Santis V., Vitale D., Guarracino F., Pellegrini F., Pietropaoli P., Singer M. (2009). Levosimendan pre-treatment improves outcomes in patients undergoing coronary artery bypass graft surgery. Br. J. Anaesth..

[63] Guarracino F., Heringlake M., Cholley B., Bettex D., Bouchez S., Lomivorotov V.V., Rajek A., Kivikko M., Pollesello P. (2018). Use of Levosimendan in Cardiac Surgery: An Update After the LEVO-CTS, CHEETAH, and LICORN Trials in the Light of Clinical Practice. J. Cardiovasc. Pharm..

[64] Bonios M.J., Terrovitis J.V., Drakos S.G., Katsaros F., Pantsios C., Nanas S.N., Kanakakis J., Alexopoulos G., Toumanidis S., Anastasiou-Nana M. (2012). Comparison of three different regimens of intermittent inotrope infusions for end stage heart failure. Int. J. Cardiol..

[65] Sterne J.A., Gavaghan D., Egger M. (2000). Publication and related bias in meta-analysis: Power of statistical tests and prevalence in the literature. J. Clin. Epidemiol..

[66] Choi S.W., Lam D.M. (2016). Funnels for publication bias—Have we lost the plot?. Anaesthesia.

[67] Zager R.A., Johnson A.C., Lund S., Hanson S.Y., Abrass C.K. (2006). Levosimendan protects against experimental endotoxemic acute renal failure. Am. J. Physiol. Ren. Physiol..

[68] Rehberg S., Ertmer C., Vincent J.L., Spiegel H.U., Köhler G., Erren M., Lange M., Morelli A., Seisel J., Su F. (2010). Effects of combined arginine vasopressin and levosimendan on organ function in ovine septic shock. Crit. Care Med..

[69] Grossini E., Molinari C., Pollesello P., Bellomo G., Valente G., Mary D., Vacca G., Caimmi P. (2012). Levosimendan protection against kidney ischemia/reperfusion injuries in anesthetized pigs. J. Pharmacol. Exp. Ther..

[70] Yilmaz M.B., Grossini E., Silva Cardoso J.C., Édes I., Fedele F., Pollesello P., Kivikko M., Harjola V.P., Hasslacher J., Mebazaa A. (2013). Renal effects of levosimendan: A consensus report. Cardiovasc. Drugs Ther..

[71] Kivikko M., Lehtonen L., Colucci W.S. (2003). Sustained hemodynamic effects of intravenous levosimendan. Circulation.

[72] Fellahi J.L., Parienti J.J., Hanouz J.L., Plaud B., Riou B., Ouattara A. (2008). Perioperative use of dobutamine in cardiac surgery and adverse cardiac outcome: Propensity-adjusted analyses. Anesthesiology.

[73] Shahin J., DeVarennes B., Tse C.W., Amarica D.A., Dial S. (2011). The relationship between inotrope exposure, six-hour postoperative physiological variables, hospital mortality and renal dysfunction in patients undergoing cardiac surgery. Crit. Care.

[74] Lindenfeld J., Albert N.M., Boehmer J.P., Collins S.P., Ezekowitz J.A., Givertz M.M., Katz S.D., Klapholz M., Moser D.K., Rogers J.G. (2010). HFSA 2010 Comprehensive Heart Failure Practice Guideline. J. Card Fail..

