# Supplementary material for: Comprehensive Comparisons among Inotropic Agents on Mortality and Risk of Renal Dysfunction in Patients Who Underwent Cardiac Surgery: A Network Meta-Analysis of Randomized Controlled Trials
Source: J Clin Med. 2021 Mar 3;10(5):1032. doi: 10.3390/jcm10051032 (PMC7959132; doi:10.3390/jcm10051032)
Supplement: Supplementary file 1 [file jcm-10-01032-s001.pdf]

SUPPLEMENTARY MATERIALS

Table S1. Detailed characteristics of each included studies

STUDY	SETTING	INTERVENTION / CONTROL	PATIENT (I/C)	LVEF BASELINE (I)[a]	LVEF BASELINE (C)[a]	KIDNEY BASELINE (I)[b]	KIDNEY BASELINE (C)[b]	INCLUSION OF PRE-EXISITNG RENAL DYSFUNCTION	AKI DEFINITION	CLINICAL END POINTS
**LASSNIGG (2000)**	Patients receiving elective cardiac surgery (CABG ± valve replacement, other) under CPB	Dopamine/ Placebo	42/40	EF> 50%,n= 32 (76%) EF< 50%,n= 10 (24%)	EF> 50%,n= 311(78%) EF< 50%,n= 9 (22%)	Scr:0.98 ± 0.23 CrCl:101 ± 35 U:72 ± 36 (ml/h)	Scr:0.96 ± 0.23 CrCl:99 ± 47 U:74 ± 43 (ml/h)	# (exclusion: Scr> 2 mg/dL)	↑Δ Scr > 0.5 mg/dL	Mortality,ICU ,AKI
**WOO (2002)**	Patients with EF<40% receiving elective cardiac surgery (CABG, combined valve and coronary surgery, isolated valve replacement) under CPB	Dopamine/ Placebo	20/22	59.1% (35-77)	56.5% (33-132)	Scr:1.08± 0.19	Scr:1.1 ± 0.16	O	NA	Mortality
**RANUCCI (2010)**	Patients receiving complex cardiac surgery requiring CPB ≥ 90 min	Fenoldopam/ Placebo	40/40	51% ± 12	55% ± 11	Scr:1.39 ± 1.65 CrCl:80 ± 41	Scr:1.08 ± 0.43 CrCl:84 ± 38	# (Preoperative CKD needing dialysis)	SCr >2 mg/dl and > baseline x2	Mortality,AKI
**AI-SHAWAF (2006)**	Patients with EF≤35% receiving CABG under CPB with LCOS after surgery	Levosimendan/ Milrinone	14/16	29% ±6	31% ±6	NA	NA	X	NA	Mortality, ICU,AKI, RRT
**ÁLVAREZ (2006)**	Patients with Postoperative LCOS (CI< 2.2 L/min/m² and PWCP > 15mmHg) after cardiac surgery with CPB	Levosimendan/ Dobutamine	25/25	NA	NA	NA	NA	X	NA	Mortality,RR T
**DE HERT(2008)**	Patients with EF≤30% receiving CABG under CPB	Levosimendan/ Milrinone	15/15	24% ± 6	27% ± 3	Scr:1.28 ± 0.49	Scr:1.05 ± 0.23	O	NA	Mortality,ICU

Table S1. Detailed characteristics of each included studies (continued)

STUDY	SETTING	INTERVENTION / CONTROL	PATIENT (I/C)	LVEF BASELINE (I)[a]	LVEF BASELINE (C)[a]	KIDNEY BASELINE (I)[b]	KIDNEY BASELINE (C)[b]	INCLUSION OF PRE-EXISITNG RENAL DYSFUNCTION	AKI DEFINITION	CLINICAL END POINTS
**JÄRVELÄ(2008)**	Aortic Valve Surgery±CABG under CPB	Levosimendan/ Placebo	12/12	50%	64%	NA	NA	-	NA	Mortality
**LEVIN (2008)**	Patients with Postoperative LCOS (CI< 2.2 L/min/m², PWCP > 16mmHg, and $ScvO_2$<60%) after CABG with CPB	Levosimendan/ Dobutamine	69/68	NA	NA	NA	NA	# (exclusion: ↑ ΔScr >50% with or without oliguria)	↑ ΔScr >50%	Mortality,ICU ,AKI,RRT
**ERIKSSON(2009)**	Patients with 3vCAD with EF≤50% receiving CABG under CPB	Levosimendan/ Placebo	30/30	36% ± 8	36% ± 8	NA	NA	-	NA	Mortality
**LEVIN (2009)**	Patients with LOS after receiving CABG under CPB	Levosimendan/ Dobutamine	126/127	NA	NA	NA	NA	X	NA	Mortality,RRT
**TRITAPEPE (2009)**	CAD receiving CABG under CPB	Levosimendan/ Placebo	52/50	44.1% ± 9.8	41.6% ± 10.7	NA	NA	# (exclusion:Scr> 1.47 mg/dL)	Scr >1.5mg/dL	ICU,AKI
**LAHTINEN(2011)**	Heart valve or combined heart valve and CABG under CPB	Levosimendan/ Placebo	99/101	EF>50%, n=77 (78%) EF30-50%, n=21 (21%) EF<30%, n=1 (1%)	EF>50%, n=73 (73%) EF30-50%, n=24 (24%) EF<30%, n=3 (3%)	Scr: 1.02 ± 0.2	Scr: 1.05 ± 0.26	O	↑ ΔScr >50%, or Scr > 2 x ULN, or RRT	Mortality,AKI ,RRT
**LEPPIKANGAS(2011)**	Patients with EF <50% or LVH >12mm receiving AVR with CABG under CPB	Levosimendan/ Placebo	12/12	63% ± 9	69% ± 9	NA	NA	-	NA	Mortality

Table S1. Detailed characteristics of each included studies (continued)

STUDY	SETTING	INTERVENTION / CONTROL	PATIENT (I/C)	LVEF BASELINE (I)[a]	LVEF BASELINE (C)[a]	KIDNEY BASELINE (I)[b]	KIDNEY BASELINE (C)[b]	INCLUSION OF PRE-EXISITNG RENAL DYSFUNCTION	AKI DEFINITION	CLINICAL END POINTS
**LEVIN (2012)**	Patients with EF <25% receiving CABG under CPB	Levosimendan/ Placebo	127/125	17.56% ± 3.24	18.62% ± 2.12	GFR:71.42 ± 4.23	GFR:73.55 ± 3.66	# (exclusion: ↑ ΔScr >50% with or without oliguria or requiring dialysis.)	↑ ΔScr >50%	Mortality,AKI ,RRT
**LOMIVOROTOV (2012)**	Patients with EF <35% receiving CABG under CPB	Levosimendan + IABP/ IABP(Placebo)	30/30	31% (28-32)	30% (29-33)	NA	NA	Δ	NA	Mortality,RRT
**RISTIKANKARE (2012)**	Patients with EF <50% or acute ischemic CHF receiving CABG under CPB	Levosimendan/ Placebo	30/30	36% ± 8	36% ± 8	Scr:0.82 ± 0.18 GFR:103 ± 26	Scr:0.81 ± 0.27 GFR:109 ± 36	# (exclusion: predialysis or end-stage CKD)	↓Δ eGFR > 25%	Mortality,AKI
**BAYSAL(2014)**	Patient with with EF≤45% and MR ± CAD receiving MV surgery under CPB	Levosimendan/ Placebo	64/64	NA	NA	Scr:1.0 (0.60-2.60)	Scr:1.1(0.50-2.60)	# (exclusion:AKI or CKD or Scr> 1.5 mg/dL)	↑ΔScr > 0.3mg/dl or ↑ΔScr >25%	Mortality,ICU ,AKI,RRT
**ERB (2014)**	Patients with EF≤30% receiving CABG under CPB	Levosimendan/ Placebo	17/16	22.0% ± 4.5	22.4% ± 5.5	NA	NA	Δ	NA	Mortality,ICU ,RRT
**SHAH (2014)**	Patients with CAD and EF≤30% receiving off-pump CABG	Levosimendan/ Placebo	25/25	22.45% ± 4.06	22.56% ± 3.41	Scr:1.21 ± 0.03	Scr:1.20 ± 0.02	# (exclusion:renal dysfunction)	↑ΔScr >50%	Mortality,AKI , RRT
**SHARMA (2014)**	Patients with CAD and EF < 30% and ischemic severe MR receiving CABG and MV repair under CPB	Levosimendan/ Placebo	20/20	23.55% ± 4.87	22.55% ± 0.92	Scr1.04 ± 0.4	Scr:0.9 ± 0.19	# (exclusion:Scr> 1.5 mg/dL)	↑ΔScr >50%	Mortality,ICU ,AKI,RRT

Table S1. Detailed characteristics of each included studies (continued)

STUDY	SETTING	INTERVENTION / CONTROL	PATIENT (I/C)	LVEF BASELINE (I)[a]	LVEF BASELINE (C)[a]	KIDNEY BASELINE (I)[b]	KIDNEY BASELINE (C)[b]	INCLUSION OF PRE-EXISITNG RENAL DYSFUNCTION	AKI DEFINITION	CLINICAL END POINTS
**ANASTASIADIS (2016)**	Patients with CAD and EF ≤ 40% receiving CABG under minimally invasive extracorporeal circulation	Levosimendan/ Placebo	16/16	35.7% ±4.9	37.5% ±3.4	NA	NA	# (exclusion:AKI or CKD or Scr> 2 mg/dL)	Scr> 2 mg/dl or >baseline x2	Mortality,ICU ,AKI
**CHOLLEY (2017)**	Patients with EF ≤ 40% receiving CABG under CPB ± valve surgery	Levosimendan/ Placebo	167/168	EF 30-40%, n=134 (80%) EF <30%, n=33 (20%)	EF 30-40%, n=129 (77%) EF<30%, n=39 (23%)	CrCl<50,n=36 (22%) CrCl>85,n=60 (36%) 50<CrCl<85, n=71 (42%)	CrCl<50,n=31 (18%) CrCl>85,n=58 (35%) 50<CrCl<85, n=79 (47%)	# (exclusion: CrCl <30 mL/min)	NA	Mortality,ICU ,RRT
**LANDONI (2017)**	Patients receiving CABG with pre-op cardio-vascular dysfunction (EF ≤ 25%, pre-op IABP), or IABP or high-dose inotropics after surgery	Levosimendan/ Placebo	248/258	EF Median 50% (37–59) EF <25%, n=11 (4.6%) EF 25-40%, n=53 (22.3%) EF >40%, n=174 (73.1%)	EF Median 50% (40–60) EF <25%, n=11(4.4%) EF 25–40%, n=43 (17.1%) EF >40%, n=197 (78.5%)	Scr:1.15 ± 0.96	Scr:1.20 ± 0.96	# (exclusion: need to receipt of a kidney or liver transplant)	RIFLE criteria	Mortality,ICU ,AKI,RRT
**MEHTA (2017)**	Patients with EF ≤ 35% receiving cardiac surgery under CPB	Levosimendan/ Placebo	428/421	26 (24-32)	27 (22-31)	NA	NA	# (exclusion: eGFR < 30 mL/min/1.73m^2)	NA	Mortality,ICU ,AKI,RRT
**SHI(2006)**	Patients with preoperative left ventricule diastolic dysfunction receiving CABG under CPB	Milrinone / Placebo	25/24	50% ± 14	50% ± 14	NA	NA	-	NA	Mortality
**COUTURE(2007)**	Patients with preoperative left ventricule diastolic dysfunction receiving CABG under CPB	Milrinone / Placebo	25/25	51% ± 15	50% ± 13	NA	NA	X	NA	Mortality,ICU ,AKI

Table S1. Detailed characteristics of each included studies (continued)

STUDY	SETTING	INTERVENTION/ CONTROL	PATIENT (I/C)	LVEF BASELINE (I)[a]	LVEF BASELINE (C)[a]	KIDNEY BASELINE (I)[b]	KIDNEY BASELINE (C)[b]	INCLUSION OF PRE-EXISITNG RENAL DYSFUNCTION	AKI DEFINITION	CLINICAL END POINTS
**ARBEUS(2009)**	Patients with with EF>30% receiving elective CABG under CPB	Milrinone / Placebo	22/22	59% ± 12	63% ± 9	NA	NA	-	NA	Mortality
**JEBELI(2010)**	Patients with with EF < 35% receiving elective CABG under CPB	Milrinone / Placebo	35/35	31.8% ± 3.2	34.5% ± 1.4	NA	NA	-	NA	Mortality,ICU
**HADADZADEH (2013)**	Patients with LVEF < 35% undergoing off pump CABG	Milrinone / Placebo	40/40	29.02% ± 5.46	28.62% ± 5.65	NA	NA	X	NA	Mortality,ICU ,AKI

The data are presented as mean±SD. AKI: Acute kidney injury; CKD: Chronic kidney disease

[a] n=patient number (total %) ; [b] Scr(mg/dl), CrCl(ml/min),GFR(ml/min/1.73m^2)

I= intervention; C= control; LVEF= left ventricular ejection fraction; ICU= intensive care unit; AKI= acute kidney injury; RRT= renal replacement therapy

Inclusion of pre-exisitng renal dysfunction (-/O/Δ/#/X): - means that the study only had mortality or ICU outcome so there was no data on kidney function; O means that the study didn't state clearly inclusion criteria but patients had normal kidney function according to baseline data; Δ means that the study didn't state clearly inclusion criteria but only some patients with mild to moderate renal dysfunction may have been enrolled (i.e. inclusion of mixed population with and without renal dysfunction); # means that the study inclusion criteria stated that only the patients with normal renal function were enrolled; X means that the study didn't provide sufficient information about patients' renal function.

Table S2. P-score analyses for the evaluated outcomes

Drugs	Outcome			
	Mortality	AKI	RRT	ICU
**Levosimendan**	90%	75%	90%	87%
**Placebo**	64%	47%	55%	46%
**Milrinone**	50%	47%	39%	55%
**Dopamine**	50%	26%	-	62%
**Dobutamine**	29%	11%	16%	2%
**Fenoldopam**	17%	93%	-	-

Table S3. Consistency analyses of each outcome by node-split model

**Comparisons**	**Direct**	**Indirect**	**z**	**p-value**
**Mortality (OR)**				
**Levosimendan v.s. Milrinone**	0.3919	0.6158	-0.3544	0.7230
**Levosimendan v.s. Placebo**	0.7415	0.4719	0.3544	0.7230
**Milrinone v.s. Placebo**	1.2042	1.8921	-0.3544	0.7230
**The incidence of AKI (OR)**				
**Levosimendan v.s. Milrinone**	0.3667	0.8083	-0.6178	0.5367
**Levosimendan v.s. Placebo**	0.6164	0.2796	0.6178	0.5367
**Milrinone v.s. Placebo**	0.7626	1.6812	-0.6178	0.5367
**ICU (MD)**				
**Levosimendan v.s. Milrinone**	-2.2620	-0.4556	-0.3234	0.7464
**Levosimendan v.s. Placebo**	-0.5448	-2.3512	0.3234	0.7464
**Milrinone v.s. Placebo**	-0.0892	1.7173	-0.3234	0.7464

p-value less than 0.05 represented presence of inconsistency. * Denotes p-value < 0.05.

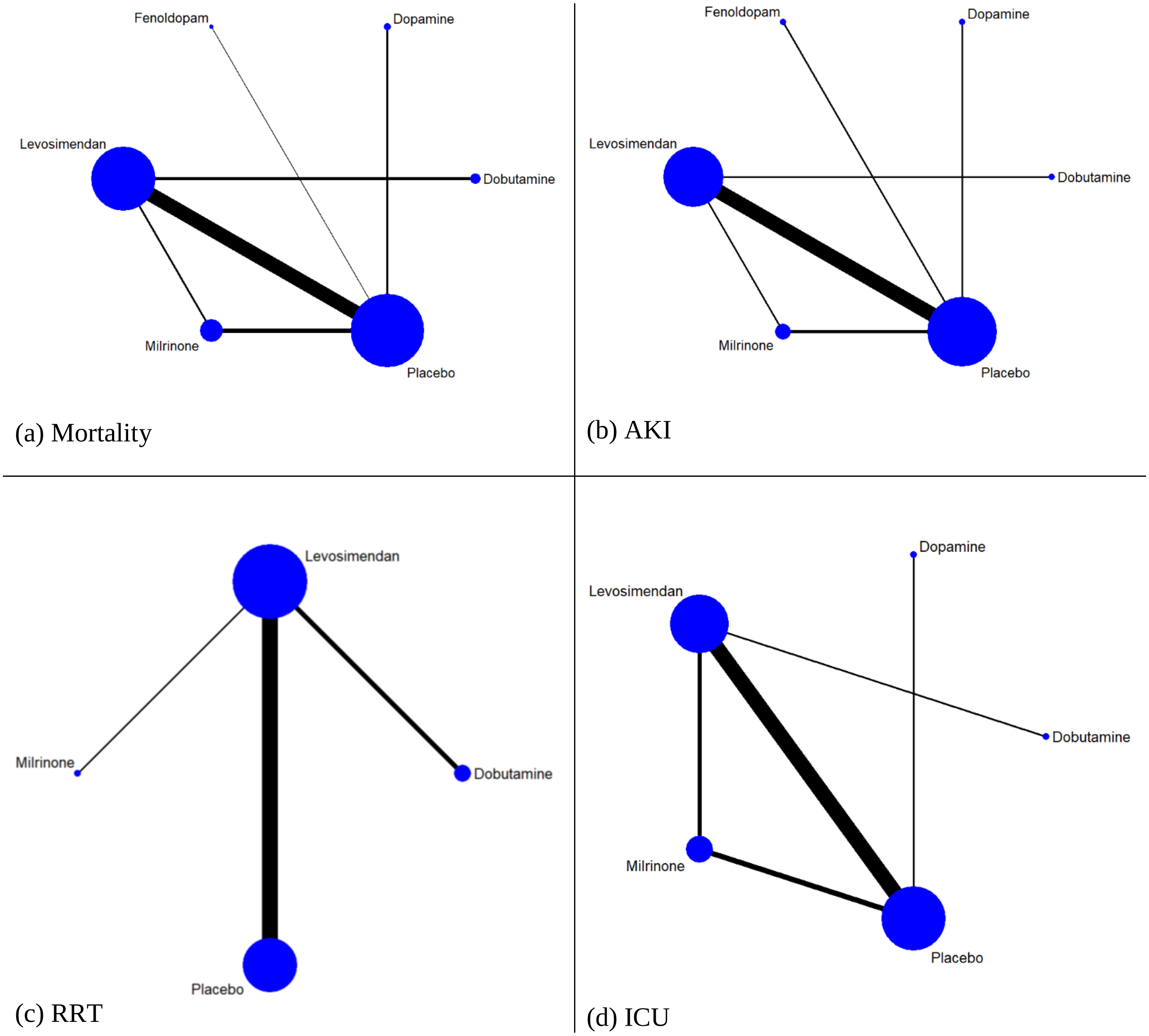

Figure S1: Network plot for included therapies.

The solid line represents as the direct comparisons. The thickness of the line represents the number of included trials; the size of the node represents the number of trials that included the drug.

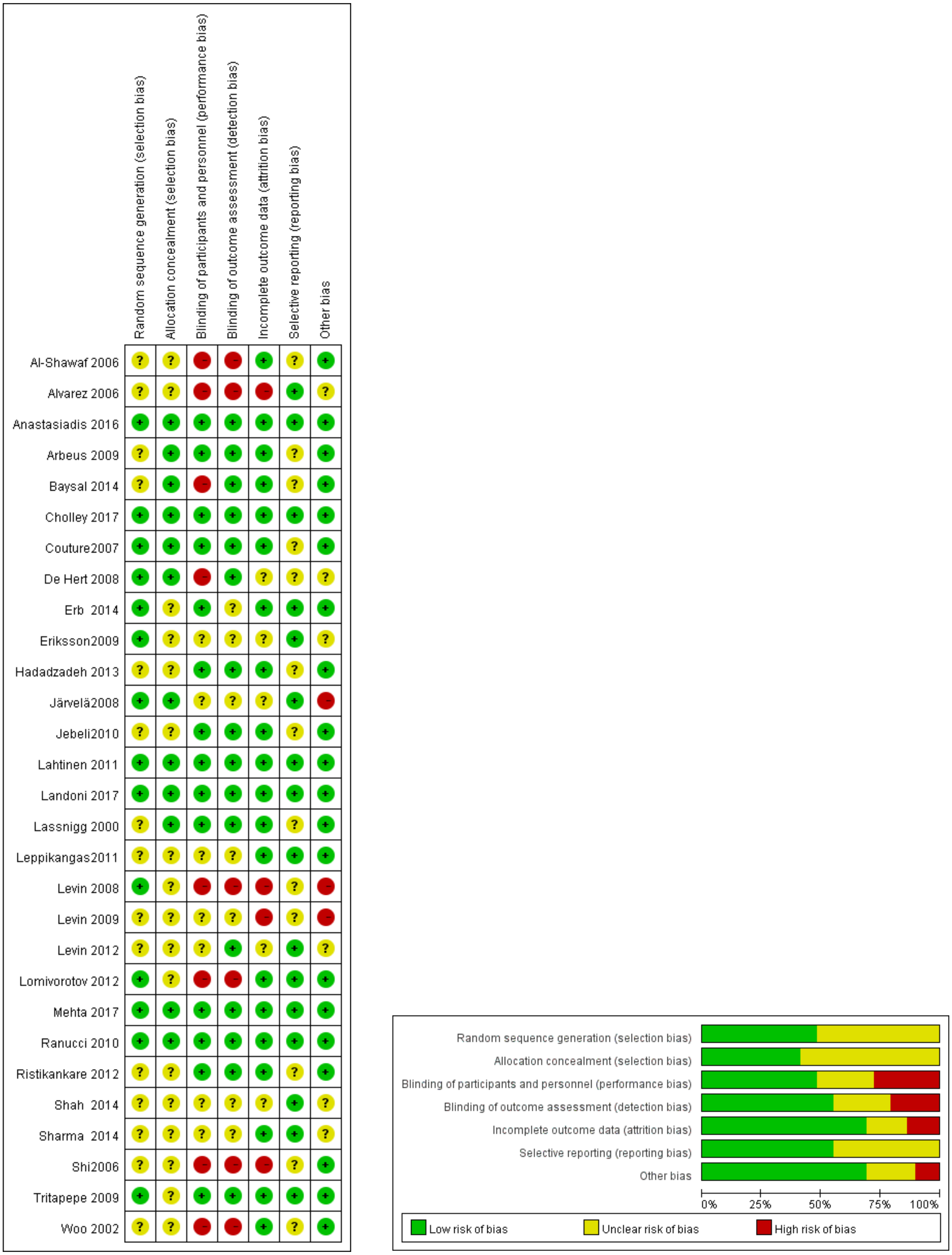

(a) **(b)**

Figure S2. Risk of bias. (a) Risk of bias summary: The judgements about each risk of bias item for each included study. (b) Risk of bias graph: The judgements about each risk of bias item reflected s percentages across all included studies.

(a)Mortality

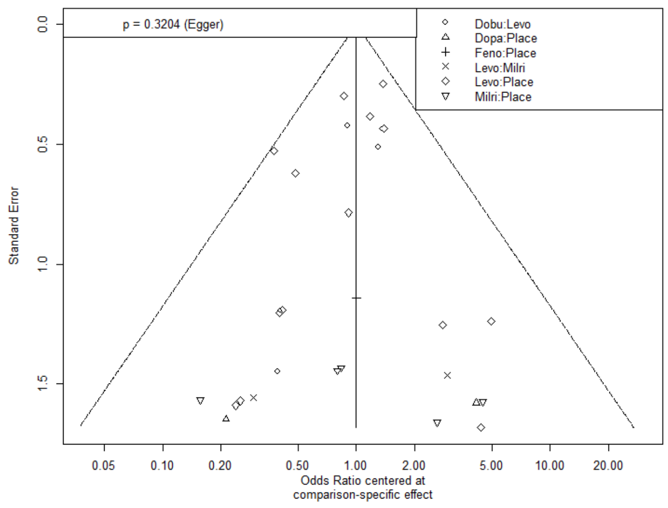

(b)AKI

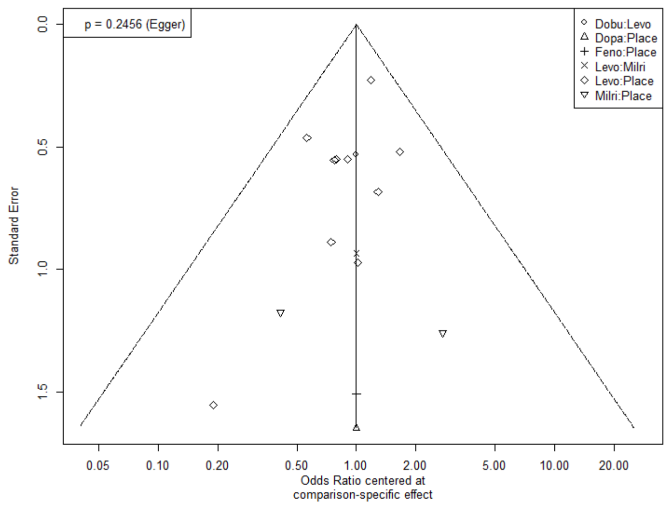

(c)RRT

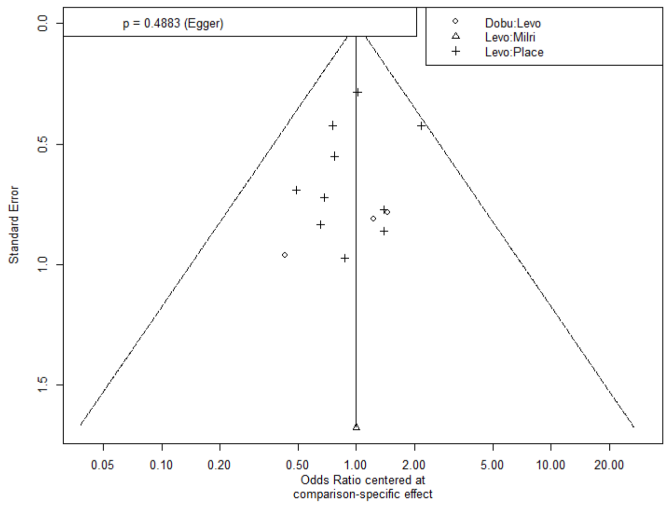

(d)ICU

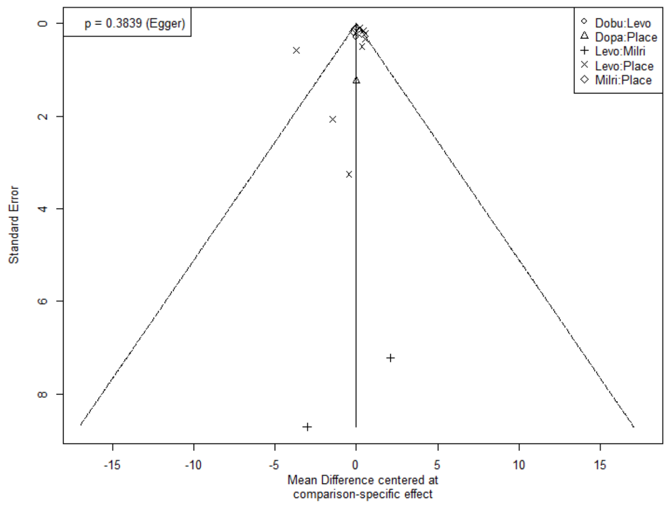

Figure S3. Funnel plot for each outcome. Each dot reflected a study; the vertical axis reflected the sample size or standard error and x-axis reflected the effect size of each study. Large studies distribute in the top of the plot, and smaller studies scatter toward the bottom of the plot. Dobu= Dobutamine; Dopa= Dopamine; Dopexa= Dopexamine; Feno= Fenoldopam; Levo= Levosimendan; Milri= Milrinone;

Study or Subgroup	Levosimendan Events	Levosimendan Total	Control Events	Control Total	Weight	Odds Ratio M-H, Random, 95% CI	Year
**1.2.1 Preoperatively**							
Järvelä2008	3	12	1	12	1.2%	3.67 [0.32, 41.59]	2008
Eriksson2009	0	30	2	30	0.8%	0.19 [0.01, 4.06]	2009
Lahtinen 2011	12	99	12	101	9.9%	1.02 [0.44, 2.40]	2011
Leppikangas2011	1	12	0	12	0.7%	3.26 [0.12, 88.35]	2011
Ristikankare 2012	0	30	2	30	0.8%	0.19 [0.01, 4.06]	2012
Lomivorotov 2012	2	30	1	30	1.2%	2.07 [0.18, 24.15]	2012
Levin 2012	5	127	16	125	6.7%	0.28 [0.10, 0.79]	2012
Sharma 2014	1	20	3	20	1.3%	0.30 [0.03, 3.15]	2014
Shah 2014	1	25	3	25	1.3%	0.31 [0.03, 3.16]	2014
Erb 2014	4	17	5	16	3.0%	0.68 [0.15, 3.16]	2014
Anastasiadis 2016	0	16	2	16	0.7%	0.18 [0.01, 3.97]	2016
Mehta 2017	20	428	30	421	21.3%	0.64 [0.36, 1.14]	2017
Cholley 2017	14	167	16	168	12.8%	0.87 [0.41, 1.84]	2017
**Subtotal (95% CI)**		**1013**		**1006**	**61.8%**	**0.67 [0.48, 0.95]**	
Total events	63		93				
Heterogeneity: Tau² = 0.00; Chi² = 10.68, df = 12 (P = 0.56); I² = 0%							
Test for overall effect: Z = 2.28 (P = 0.02)							
**1.2.2 Intraoperatively**							
Landoni 2017	38	248	39	258	30.7%	1.02 [0.63, 1.65]	2017
**Subtotal (95% CI)**		**248**		**258**	**30.7%**	**1.02 [0.63, 1.65]**	
Total events	38		39				
Heterogeneity: Not applicable							
Test for overall effect: Z = 0.06 (P = 0.95)							
**1.2.3 Postoperatively**							
Al-Shawaf 2006	1	14	1	16	0.9%	1.15 [0.07, 20.34]	2006
Alvarez 2006	1	21	1	20	0.9%	0.95 [0.06, 16.29]	2006
De Hert 2008	0	15	3	15	0.8%	0.12 [0.01, 2.45]	2008
Baysal 2014	4	64	10	64	4.9%	0.36 [0.11, 1.22]	2014
**Subtotal (95% CI)**		**114**		**115**	**7.4%**	**0.41 [0.15, 1.11]**	
Total events	6		15				
Heterogeneity: Tau² = 0.00; Chi² = 1.55, df = 3 (P = 0.67); I² = 0%							
Test for overall effect: Z = 1.76 (P = 0.08)							
**Total (95% CI)**		**1375**		**1379**	**100.0%**	**0.74 [0.56, 0.96]**	
Total events	107		147				
Heterogeneity: Tau² = 0.00; Chi² = 15.56, df = 17 (P = 0.56); I² = 0%							
Test for overall effect: Z = 2.24 (P = 0.03)							
Test for subgroup differences: Chi² = 3.30, df = 2 (P = 0.19), I² = 39.4%							

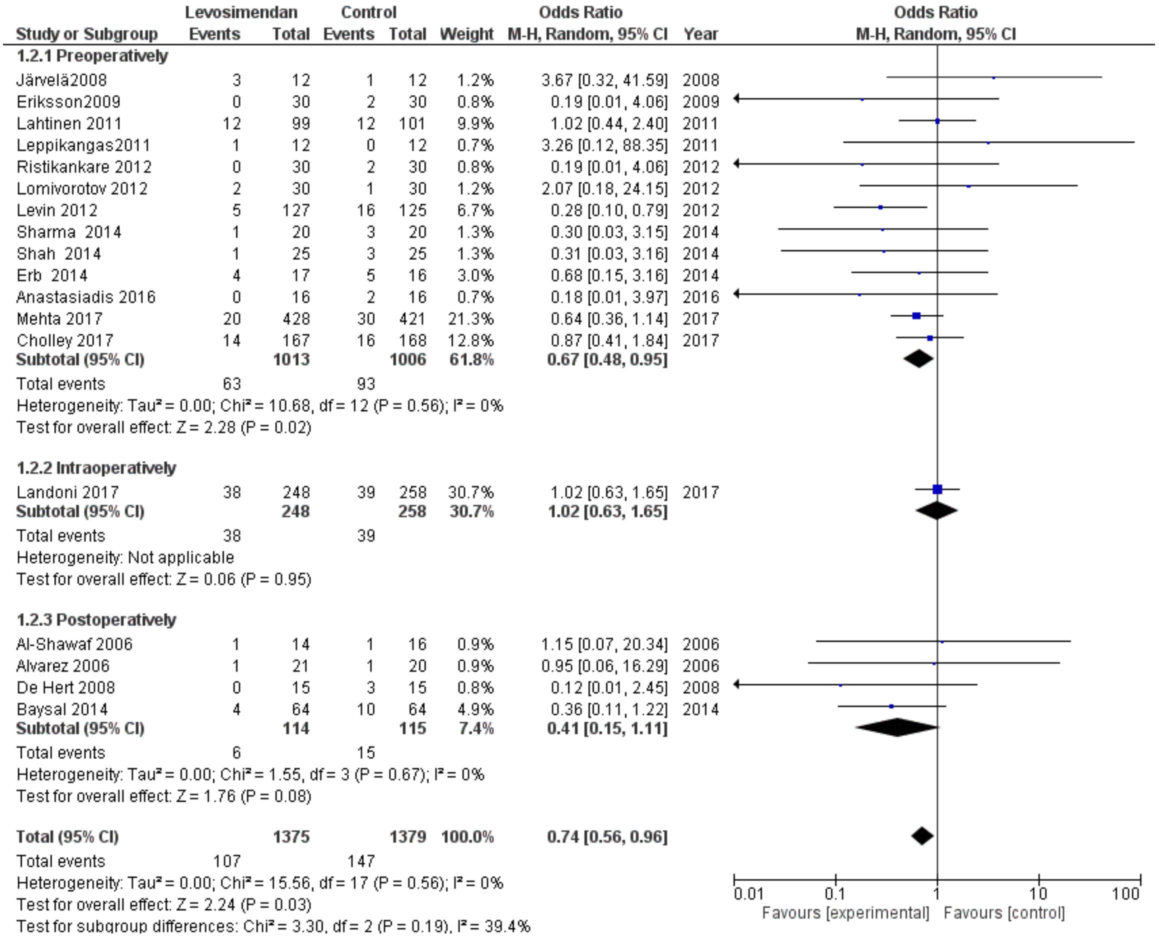

Figure S4. Subgroup analyses for mortality by administration timing

Data presented as odd ratio (OR) with 95% confidence interval (CI). Square represented estimated OR in each study and its size reflected the sample size; 95% CI is represented horizon lines. P-value of subgroup difference < 0.1 means having significant subgroup effect.

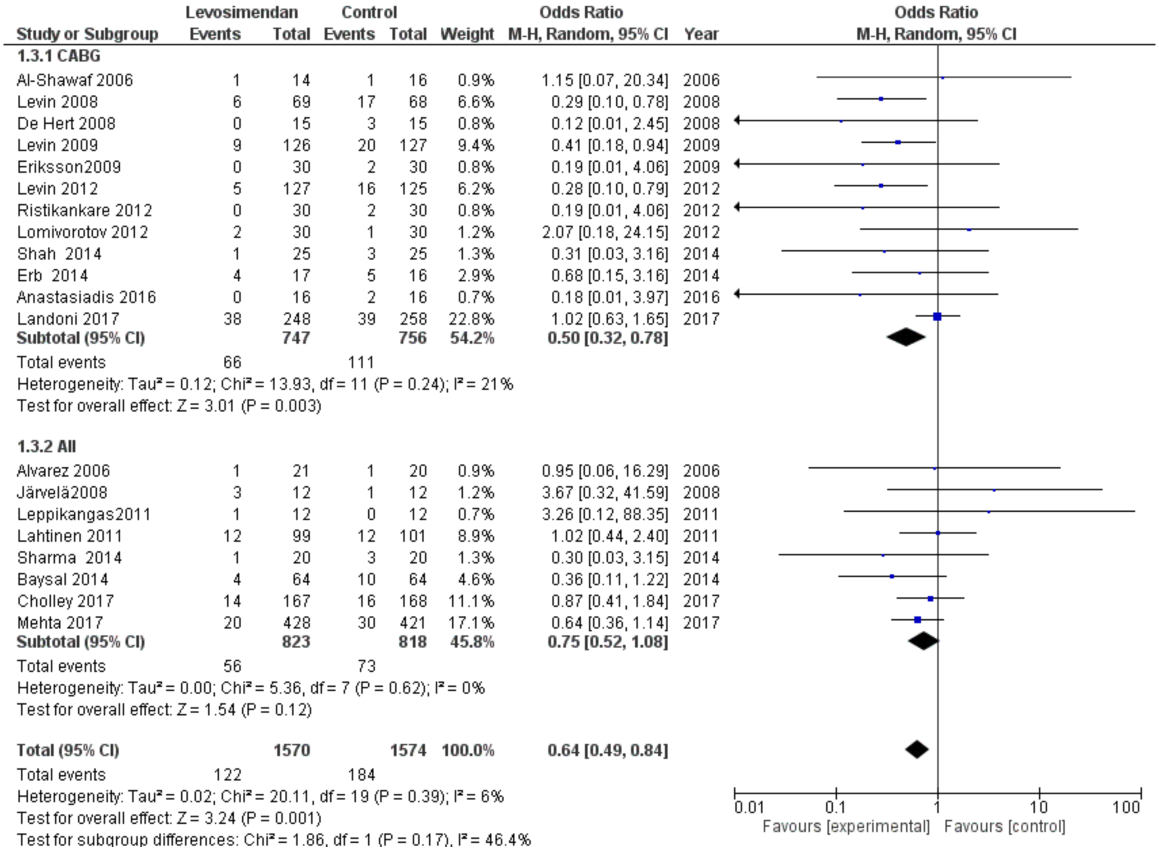

Figure S5. Subgroup analyses for mortality by operation method.

Data presented as odd ratio (OR) with 95% confidence interval (CI). Square represented estimated OR in each study and its size reflected the sample size; 95% CI is represented horizon lines. P-value of subgroup difference < 0.1 means having significant subgroup effect.

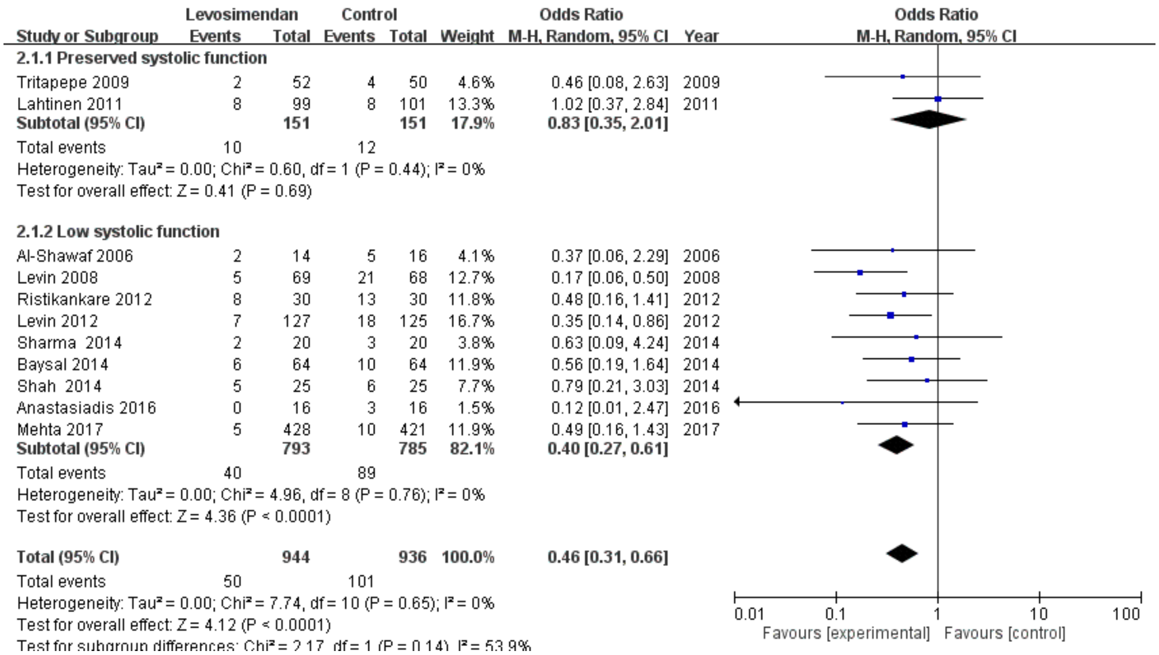

Figure S6. Subgroup analyses for AKI by systolic function.

Data presented as odd ratio (OR) with 95% confidence interval (CI). Square represented estimated OR in each study and its size reflected the sample size; 95% CI is represented horizon lines. P-value of subgroup difference < 0.1 means having significant subgroup effect.

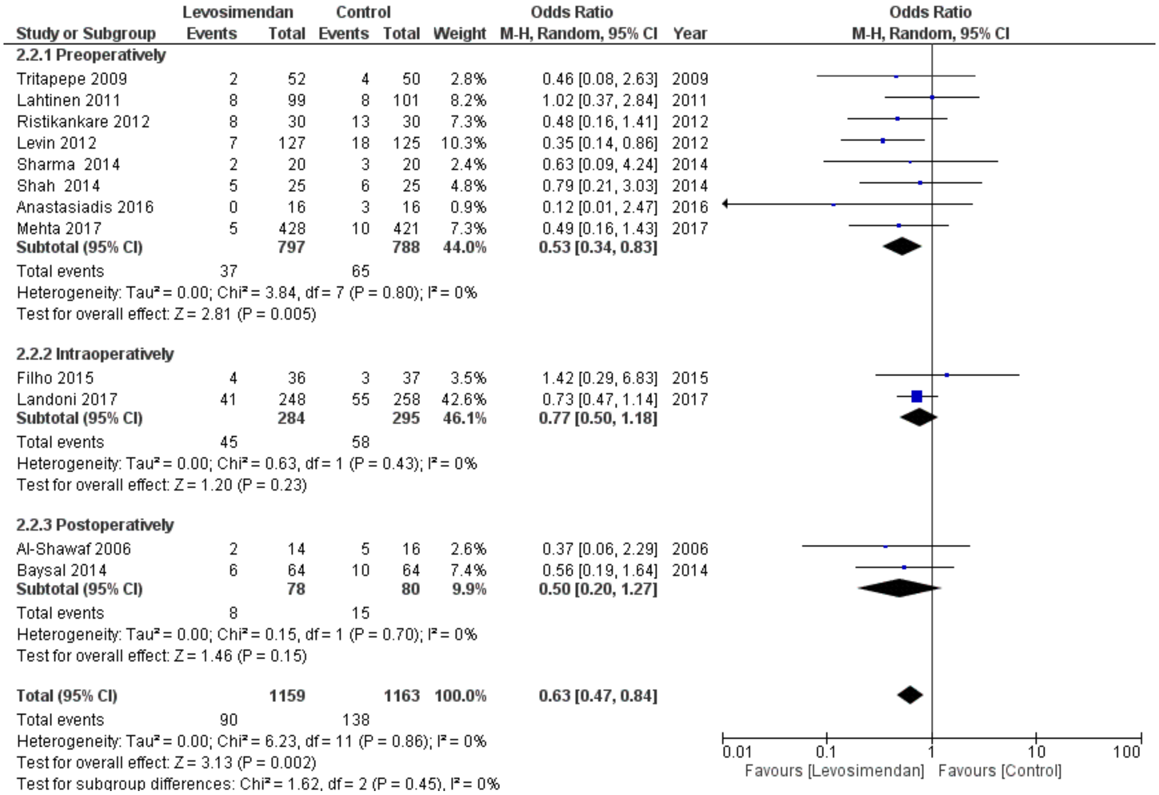

Figure S7. Subgroup analyses for AKI by administration timing.

Data presented as odd ratio (OR) with 95% confidence interval (CI). Square represented estimated OR in each study and its size reflected the sample size; 95% CI is represented horizon lines. P-value of subgroup difference < 0.1 means having significant subgroup effect.

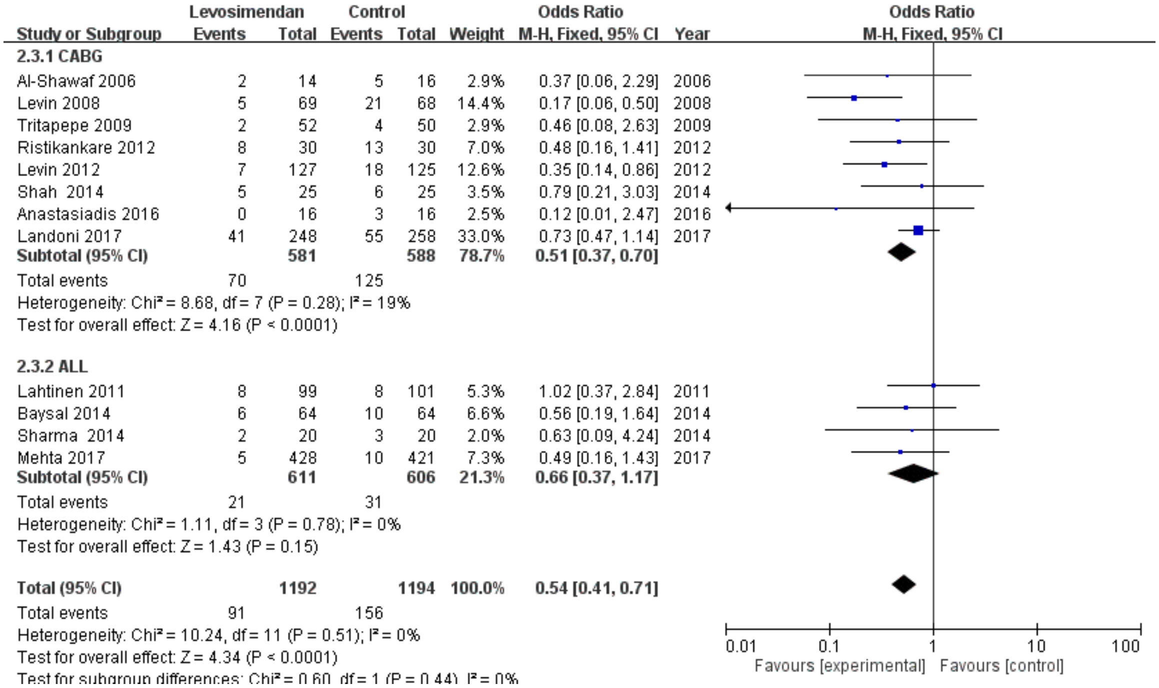

Figure S8. Subgroup analyses for AKI by operation method.

Data presented as odd ratio (OR) with 95% confidence interval (CI). Square represented estimated OR in each study and its size reflected the sample size; 95% CI is represented horizon lines. P-value of subgroup difference < 0.1 means having significant subgroup effect.
